# Application of 3D Zernike descriptors to shape-based ligand similarity searching

**DOI:** 10.1186/1758-2946-1-19

**Published:** 2009-12-17

**Authors:** Vishwesh Venkatraman, Padmasini Ramji Chakravarthy, Daisuke Kihara

**Affiliations:** 1 Department of Biological Sciences, Purdue University, 915 West State Street, West Lafayette, IN 47907, USA; 2 Markey Center for Structural Biology, Purdue University, 915 West State Street, West Lafayette, IN 47907, USA; 3 Department of Electrical and Computer Engineering, Purdue University, 915 West State Street, West Lafayette, IN 47907, USA; 4 Department of Computer Science, Purdue University, 915 West State Street, West Lafayette, IN 47907, USA

## Abstract

**Background:**

The identification of promising drug leads from a large database of compounds is an important step in the preliminary stages of drug design. Although shape is known to play a key role in the molecular recognition process, its application to virtual screening poses significant hurdles both in terms of the encoding scheme and speed.

**Results:**

In this study, we have examined the efficacy of the alignment independent three-dimensional Zernike descriptor (3DZD) for fast shape based similarity searching. Performance of this approach was compared with several other methods including the statistical moments based ultrafast shape recognition scheme (USR) and SIMCOMP, a graph matching algorithm that compares atom environments. Three benchmark datasets are used to thoroughly test the methods in terms of their ability for molecular classification, retrieval rate, and performance under the situation that simulates actual virtual screening tasks over a large pharmaceutical database. The 3DZD performed better than or comparable to the other methods examined, depending on the datasets and evaluation metrics used. Reasons for the success and the failure of the shape based methods for specific cases are investigated. Based on the results for the three datasets, general conclusions are drawn with regard to their efficiency and applicability.

**Conclusion:**

The 3DZD has unique ability for fast comparison of three-dimensional shape of compounds. Examples analyzed illustrate the advantages and the room for improvements for the 3DZD.

## Background

A crucial step in early phase drug discovery is the identification of promising drug leads *i.e*. those of pharmacological interest. A guiding premise in this stage is that of the *similarity property principle *[1,2] which states that similar structures are likely to have similar properties (although exceptions to this rule do exist [3,4]). Proceeding along these lines, similarity based virtual screening efforts [5] look for nearest neighbours for a given query structure. The output of the screen is in the form of a sorted list, where top-ranking molecules are selected to undergo further processing.

As compound databases can hold millions of structures (spanning a large chemical space), the application of such approaches requires suitable molecular representations that aid rapid screening. An additional requirement is that of a numerical measure that quantifies the similarity between the compounds. Popular descriptions include fingerprints that encode the two-dimensional molecular structure as a bit string where each value indicates the presence or absence of a desired attribute (*e.g. *a substructural fragment) [6,7]. Similarities between the ligands can then be obtained using the Tanimoto score [8] which accounts for the number of bits shared by the fingerprints. While these descriptors are extremely efficient and easy to calculate they have still some limitations [9]. A number of three-dimensional (3D) similarity methods [10-12] have therefore been developed to investigate if the 3D structure information improves over the existing descriptors.

While a number of techniques for 3D molecular comparison have been proposed [1,13,14], in this article, we focus on similarity-based virtual screening using molecular shape [15-17] as the key feature for discrimination. Shape is known to play an important role in molecular-recognition, with previous studies demonstrating successful applications to virtual screening experiments [16,18]. However, identifying suitable encodings based on shape are far from trivial [18-21], which pose significant hurdles in their application to fast screening of compound databases.

In order to facilitate efficient comparisons, several representations of shape have been proposed ranging from those based on moments [22] and surfaces [21,23] to grid-based approaches [24] (see Putta & Beroz [15] for a comprehensive review). A well-established method is that of ROCS (Rapid Overlay of Chemical Structures)[16] that describes the molecule as a set of atom-centered Gaussians [25]. Shape similarity scores are then evaluated in terms of the rigid body overlap volume with comparison timings in the milliseconds range. Goldman and Wipke [26], on the other hand, divide the molecular surface [27] into a series of patches (2Å radius) centered on a set of critical points [28] with each patch defined by a geometrically invariant descriptor (the principal curvatures, normals, and the shape index). Points with similar geometric signatures are identified, based on which a transformation can be calculated. Proceeding along the same lines, SURFCOMP [21] uses a graph matching to identify correspondences between shape (local curvature) critical points of the molecular surfaces being compared. Although the superimpositions found using the above two methods were found to be reasonably accurate, pairwise comparisons took more than a minute, which limits their large scale application.

Spherical harmonics based representations [29,30] have been further applied to comparing shapes of ligand binding sites [31] and as geometric filters for virtual high throughput screening [32]. The use of spherical harmonics allows the surface information to be encoded in a compact form as an orthonormal one-dimensional (1D) vector of floating numbers rendering it amenable to fast comparison. However, for the molecules to be compared, they have to be placed in a standard frame of reference. This has been shown to be error-prone and hence may result in the decreased performance of the descriptor [33,34]. Consequently, descriptors that obviate the need for any pre-alignment have been the focus in a number of studies. Shape signatures [18,35], for example, produce a 1D representation of the ligand or receptor site by ray-tracing the molecular volume. The geometric information is encoded as a probability distribution which enables fast comparisons using the shape histograms. The signatures can be further extended to incorporate other properties such as electrostatics. Another method that captures shape independent of orientation is the Ultrafast Shape Recognition (USR) scheme [22]. In this technique, the 3D molecular shape is represented as a set of statistical moments, generated from all atom distance distributions that are calculated with respect to preselected reference locations.

More recently, a number of articles [36-41] have advocated the use of 3D Zernike invariants as descriptors for shape comparison. An extension of spherical harmonics, the 3D Zernike descriptors (3DZD) have favourable features such as orthonormality and compactness. More importantly, the 3DZD are also invariant to transformation (see section on computational details), and thus the pre-alignment step is no longer required. Represented by a 1D set of numbers (subject to a specified order of expansion), the 3DZD have facilitated rapid screening of protein databases [38], discrimination of proteins based on the electrostatic potential [40] and for analyzing shapes of ligand binding pockets [34,37].

In this paper, we examine the efficacy of the 3DZD as a tool for shape similarity based virtual screening. Due to its compact representation, 3DZD enable fast comparison of compounds, which is a key property of virtual screening methods given the fast growing size of molecular databases. The performance is compared with several other methods, USR [22], SIMCOMP [42], EVA [43], Unity2D [44], Molprint-2D [7], and MACCS [6]. These approaches and the metrics used for evaluation are briefly described in the next section. Three datasets were used: the first one is a set of 47 diverse odour compounds (divided into seven classes) taken from a previous study by Takane and Mitchell [43], while the second one is the Directory of Useful Decoys (DUD) dataset [45,46], that contains 13 targets with 66013 compounds. The last dataset includes 42,689 anti-HIV inhibitors [47] categorized into active, inactive, and moderately active. With the first dataset, we test the ability of the methods to classify compounds, while the second dataset is employed to examine the ability to rank actives among decoys. In addition, the third dataset is used to simulate actual virtual screening process against a large pharmaceutical database. Results evaluated with respected to the datasets are assessed in terms of several evaluation metrics. Reasons for the failure of the shape based methods for specific cases are investigated. Based on the results for the three datasets, general conclusions are drawn with regard to their efficiency and applicability with suggestions for future work.

## Computational and Experimental Details

### Methods for structure comparison

Computational approaches for ligand screening used in this study are briefly introduced here. For further details, please refer to the cited articles.

### 3D Zernike Descriptors

The 3D Zernike functions [39] are given by(1)

where  are complex valued spherical harmonics expressed in terms of spherical coordinates (θ, φ), *n, l, m *are integers such that |*m*| ≤ *n *and *n *- |*m*| is even and *R*_*nl*_*(r) *represents orthogonal radial polynomials. Given a 3D shape function *f*(*x*): *x *∈ *R*^3^, the Zernike moments are the projection of the shape function onto these orthogonal basis functions. For an order *n *they can be expressed as a linear combination of scaled geometrical moments (to fit a unit sphere)(2)

The moments however are not rotationally invariant but as rotations do not change the magnitudes of the functions, the invariant features are expressed in terms of the norms . Translational invariance is obtained by fixing the coordinate system with the origin coinciding with the spatial center of the molecule. From mathematical point of view, this procedure is proven to compute the identical descriptor for an object regardless of the positioning of the object in the space [36,48]. However, in practice some variance may be caused due to numerical errors and the voxelization step of the object necessary to represent the shape of the object. In our previous paper, we examined the variance caused by rotation [38]. An advantage of the 3DZD is that it can also describe non-star-like shapes. This is a limiting factor for spherical harmonics as they can only model single-valued surfaces [34].

Extraction of moments starts with the generation of a suitable molecular surface. In this study, the Connolly surface [27] definition has been used. Unlike the spherical harmonics which are calculated with respect to a spherical grid, the 3D Zernike formulation uses a rectangular grid (voxelization) to compute the geometrical moments. Moments of orders ranging from *n *= 4 to *n *= 14 have been examined in this study, with each molecule represented as a 1D floating point vector of  numbers when n is an even number [34]. Three distance measures have been used to compare structures based on their 3DZD representations (*X *and *Y*). These include the Euclidean distance (*D*_*E*_), the Pearson correlation coefficient (*r*) and the third based on a scaled Manhattan distance (*D*_*M*_)[22,48]. While the first two measures are commonly used in similarity searching, the third metric is taken from an earlier article by Ballester and Richards [22].(3)

Note that D_E _is 0 for two identical molecules, while the correlation coefficient and D_M _give the value of 1 for that case.

### Ultrafast Shape Descriptor

A purely atom-based approach, ultrafast shape recognition (USR) uses the statistical moments (mean, standard deviation and skewness) generated from the interatomic distance distributions. The moments attempt to define shape in terms of the size, compactness and the asymmetry associated with the structure. The all-atom distance distributions are calculated with respect to four reference points: the centroid (ctd), the atom closest to the centroid (cst), the atom furthest to the centroid (fct) and the farthest atom to fct (ftf). In the original implementation, Ballester and Richards [22] had used the first three moments yielding a 12-valued descriptor.(4)

In this study, the fourth central moment kurtosis (see Equation 4) [49] has also been included, which gives a descriptor of length 16 (referred to as USR-*k*):(5)

In the original implementation of the USR, similarities between the structures being compared is then calculated based on D_M _with the score (range 0-1) given by(6)

Here, *M*_*Q *_and *M*_*D *_are the one-dimensional vectors corresponding to the query and database molecules and *N *is the length of the vector, determined by the number of statistical moments considered *i.e. N = 12 *for the first three and *N = 16 *for the first four. In addition to D_M_, in this study we also employed the Euclidean distance (D_E_) and the correlation coefficient.

### The other existing programs compared in this study

In addition, we compared with several existing programs: EVA [43], UNITY2D [44], SIMCOMP [42], Molprint-2D [7], and MACCS [6]. Below we provide a brief description of the characteristics of the methods.

The EVA (Eigen VAlue) descriptors are derived from the vibrational frequency calculations (calculated normal modes) with each molecule represented as a vector of 761 numbers [43]. In the UNITY2D [44] the molecule is encoded as a Boolean array of 922 bits (available in SYBYL 7.1) that encode the presence (1) or absence (0) of substructural features. The results of these two methods are taken from the paper by Takane & Mitchell (2004), who analyzed the odour dataset.

The program SIMCOMP [42], uses a graph matching approach to compare chemical compounds. Each molecule is represented as a two-dimensional graph [50] with atoms and bonds becoming the vertices and edges respectively. Each atom is then assigned a label based on its neighbourhood *i.e*. the atom-typing scheme takes into consideration the adjacent atoms, the type of bonds they are involved in, and whether they have an associated ring structure. In all 68 different atom types were defined that included 23 carbon atom types, 18 oxygen, 7 sulphur, 2 phosphorous, and the rest for halogens and others. The edges are labelled according to the type of bond (single, double, triple) they represent. With this representation in place, the problem of finding a match is reduced to that of identifying a maximal common subgraph (subgraph of one graph that is isomorphic to a subgraph of the other) between the two graphs being compared. For this purpose, an association graph (AG) is constructed that encodes the possible mappings between the nodes (similar atom environments) of the two graphs. Further, each vertex pair in AG is also assigned a weight, 0.5 for cases where partial matches for the same atom species with different environments were found. All the other pairs were weighted as 1 if they belonged to the same atom species and 0 otherwise. SIMCOMP adopts a clique (fully connected subgraph) detection approach (a modified version of the Bron-Kerbosch (BK) algorithm [51]), to identify common substructures among which subgraphs with the largest sum of weights are sought. As graph matching has a high time complexity, additional heuristics in the form of a minimum size of the cliques to be found and the number of recursions of the BK algorithm to be executed were introduced to speed up the matching. Based on the largest match found, a numerical measure of the similarity between the two structures S(G1, G2) was calculated as(7)

Here, *MCS *is the maximal common subgraph found and |.| represents the number of vertices in the graphs. The score depends on the sizes of the graphs and ranges between 0 and 1.

The MACCS descriptors are a set of 166 predefined structural keys that encode patterns in the molecule (such as the presence of S-S bonds, rings of size 4, presence of halogen etc).

The Molprint-2D fingerprint [7] also encodes the molecule as a binary vector by taking into consideration the atom environment (only heavy atoms) *i.e. *the counts of the types of the atoms (SYBYL atom types are used) within two bond-lengths of a central atom. The bits thus encode the presence or absence of these atom environments.

In addition, we employ a method which simply considers the number of atoms in the molecule (the atom count method). The similarity of two molecules is defined as the difference of the number of atoms. This method serves as the reference to examine the effect of using shape information by the above methods.

### Datasets

The aforementioned approaches were tested on three datasets that are chosen to demonstrate the applicability of the methods for classification and rapid screening of large databases. The first dataset was taken from an earlier study by Takane and Mitchell [43], who attempted a clustering of 47 odour compounds using the EVA descriptors. The compounds are divided into seven categories: amber (9 compounds), bitter almond (9 compounds), camphor (5 compounds), musk (11 compounds), jasmine (2 compounds), rose (5 compounds) and muguet (6 compounds).

The second data set is Directory of Useful Decoys (DUD) dataset [45]. It is derived from ZINC [52], a database of commercially available compounds for virtual screening. A subset of the DUD containing 13 targets that is more suitable for vertical screening has often been used in recent studies [46,53-55]. This subset is the result of a filter approach applied to the original DUD dataset (40 targets with about 95172 compounds) that not only removes molecules with unsuitable physicochemical properties but also resolves the problem of bias of an artificial enrichment (due to the presence of close analogues of the actives). The first step of the filtering protocol involved the generation of the seed structures for the actives (obtained from http://dud.docking.org/clusters/) using CORINA3D and refinement using MACROMODEL at pH 7.0 [56]. The decoys were also subjected to a similar refinement process. Subsequently, a filtering process is applied to retain lead like structures (Alog *P *filter of 5.5 for nuclear hormone receptors; 4.5 for others) [46]. See Table 1 for the final composition. The prepared structures for the actives and decoys for the 13 DUD targets were downloaded http://dud.docking.org/jahn/ and have been analysed in this study. For the search query, crystal structure coordinates of the same complexed ligands were taken from the DUD http://dud.docking.org/r2/dud_target_ligands.tar.gz.

**Table 1:** Breakdown of the DUD dataset.

Target	PDB	Actives	Decoys	Decoys per active
angiotensin-converting enzyme (ace)	1o86	46	1796	39.04
acetylcholinesterase (ache)	1eve	100	3859	38.59
cyclin-dependent kinase 2(cdk2)	1ckp	47	2070	44.04
cyclooxygenase-2(cox2)	1cx2	212	12606	59.46
epidermal growth factor receptor(egfr)	1m17	365	15560	42.63
factor Xa(fxa)	1f0r	64	2092	32.69
HIV reverse transcriptase(hivrt)	1rt1	34	1494	43.94
enoyl ACP reductase(inha)	1p44	57	2707	47.49
P38 mitogen activated protein(p38)	1kv2	137	6779	49.48
phosphodiesterase(pde5)	1xp0	26	1698	65.31
platelet derived growth factor receptor kinase(pdgfrb)	1t46	124	5603	45.19
tyrosine kinase SRC(src)	2src	98	5679	57.95
vascular endothelial growth factor receptor(vegfr2)	1fgi	48	2712	56.5

The third dataset from the National Cancer Institute (NCI) consists of 42,687 compounds derived from an assay measuring protection from HIV-1 infection of human CEM cells [57]. The compounds were further categorized into 423 confirmed actives (100% protection), 1,081 moderately actives (> 50% protection) and 41,185 confirmed inactives (<50% protection) yielding a ratio of 97 decoys per active. More details of the dataset is available at the website at NCI http://dtp.nci.nih.gov/docs/aids/aids_data.html. The coordinates for these structures were downloaded from http://ligand.info in the SDF format. The dataset not only resembles a pharmaceutical database but also enables the extraction of actives in a form akin to that of a typical virtual screening experiment. For cases with missing coordinates, the structures were rebuilt using CORINA [58]. Examination of the compounds in the dataset revealed that a small number of cases had disconnected components. As neither the 3DZD nor USR can currently handle such structures, we decided to choose the largest fragment to represent the compound. Following a previous work on the same dataset by von Grotthus *et al. *[59], the objective was to test the retrieval capability of the actives using the 1081 moderately actives as queries. The datasets used are available at our website, http://kiharalab.org/zernikeligand/.

### Evaluation Metrics

In order to assess the performance of the different methods used for screening, the following metrics have been used while taking into consideration the size of the datasets:

1) Clustering and the Adjusted Rand Index- For the odour dataset, results were evaluated based on the quality of the clustering obtained. Ward's hierarchical clustering [60] was done using software downloaded from http://www.let.rug.nl/kleiweg/clustering/. Starting with an initial number of clusters (say N), Ward's method merges two clusters at a time while minimizing the sum of squared errors at each step. To compare, the quality of the clusters, the adjusted Rand index [43,61] has been applied and is given by(8)

where *G*_1 _and *G*_2 _are the true and predicted partitions of the data set of size *N*, *m*_*ij *_is the number of samples in both class *i *of *G*_1 _and class *j *of *G*_2_, *m*_*i *_and *m*_*j *_are the number of samples in the ith and jth class of the partitions of *G*_1 _and *G*_2_, respectively. The index provides a numerical measure of the agreement between the original and predicted clusters and ranges between 0 for dissimilar groupings to 1 for similar ones.

2) Enrichment factor - This metric [62] describes the ratio of actives retrieved relative to the percentage of the database scanned. If *T*_*A *_be the total number of actives in a database of size *T*_*D *_and *N*_*a *_be the number of actives in the top *x *percent *N*_*x *_of the database, then the enrichment factor is given by(9)

3) BEDROC - Although frequently used, the enrichment factor has a major drawback in the form of the "early recognition problem". It does not distinguish between schemes that rank actives ranked at the top of the list from those that place them at the end. As actives ranked earlier in the list are desired, the Boltzmann enhanced discrimination of receiver operating characteristic or BEDROC [63] was proposed to evaluate the performance of ranking methods. The metric is given by(10)

where, n is the number of actives among N compounds, ri is the rank of the ith active and α is a parameter that assigns a weight towards compounds the top of the ranked list. The BEDROC metric ranges between 0 and 1 and in this study, has been calculated for α = 160.9 and α = 32.2 which corresponds to the top 1% and 5% of the relative rank accounting for 80% of the BEDROC score.

*4) Area Under Curve for Receiver Operator Characteristic (ROCAUC) *- The area under the curve metric represents the probability of a randomly chosen active being ranked higher than a randomly chosen decoy [64]. If *N *be the number of compounds with *N*_*a *_actives, and *N*_*d *_decoys the area under the ROC curve is given by(11)

where  is the number of decoys ranked above the *i*^*th *^active.

### Implementation Details

All calculations were performed on a 2.13 GHz Intel dual processor system running Linux with 8 GB RAM. Programs for the extraction and comparison of the moments based on the 3DZD and USR were written in C++. For the USR approach, the extraction of moments typically takes around 4 ms on an average. Times for the 3DZD on an average take about 1s including surface generation which is about 250 times that of the USR. However, this step needs to be performed only once and can be directly stored in a database.

Software for SIMCOMP [42] was downloaded from the KEGG website http://web.kuicr.kyoto-u.ac.jp/simcomp/. The software executable SIMCOMP which performs a pairwise comparison of two structures was used to calculate the similarities. Prior to the calculation, all structures were converted into the required KCF (KEGG Chemical Function) format using the SOAP/WSDL http://www.genome.jp/kegg/soap/ interface provided by the KEGG database.

## Results

### Odour dataset

**Figure 1:**
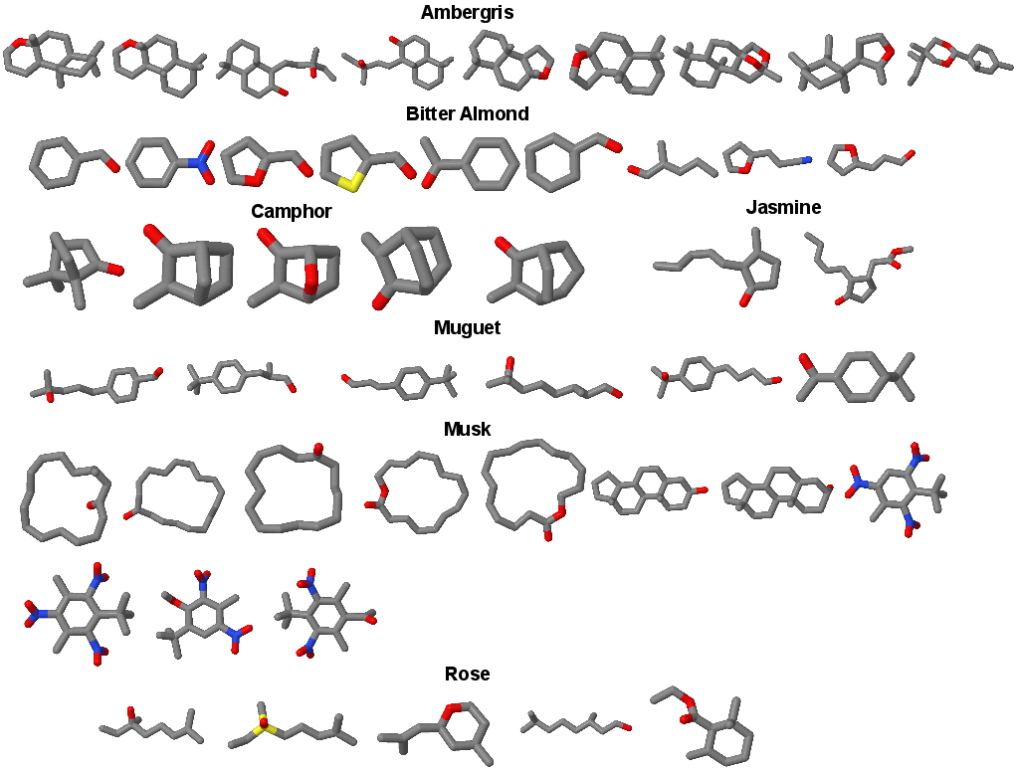
**Structures of the 47 odour compounds that are divided into seven categories: amber, bitter almond, camphor, jasmine, rose, muguet, and musk**.

Pairwise comparisons of the 47 odour compounds (Figure 1) were performed using all approaches, *i.e. *the 3DZD, USR and its kurtosis variant, SIMCOMP, EVA, UNITY2D, MACCS, and Molprint2D. The values of EVA and UNITY2D are taken from the previous study of EVA [43]. In addition, we employed a simple atom counting method as a reference, which compares the number of atoms in compounds. Since the main objective was to see if the methods could provide correct groupings of the seven fragrances, the data set was clustered into as many groups, based on the similarity matrix obtained. In order to compare the quality of the clustering against the experimentally determined classification, the adjusted Rand index, BEDROC, and ROCAUC values are calculated (Table 2).

**Table 2:** Adjusted Rand Indices, BEDROC, and AUCROC values.

Comparison Method						
Descriptors	Metric	Order	Adjusted Rand Index	BEDROC (α = 160.9)	BEDROC (α = 32.2)	ROC AUC
3DZD	Correlation coefficient	4	0.299	0.506	0.453	0.694
		6	0.256	0.590	0.494	0.708
		8	0.422	0.739	0.654	0.738
		10	0.465	0.614	0.536	0.724
		12	0.487	0.697	0.630	0.732
		14	0.442	0.697	0.618	0.733
	Euclidean	4	0.278	0.338	0.329	0.680
		6	0.278	0.526	0.446	0.704
		8	0.357	0.678	0.621	0.738
		10	0.395	0.594	0.553	0.722
		12	0.487	0.658	0.610	0.730
		14	0.372	0.717	0.622	0.743
	Manhattan	4	0.270	0.318	0.329	0.686
		6	0.260	0.484	0.427	0.703
		8	0.328	0.698	0.619	0.732
		10	0.408	0.591	0.619	0.736
		12	0.393	0.637	0.591	0.732
		14	0.213	0.656	0.598	0.748
USR	Correlation coefficient	12	0.213	0.697	0.617	0.695
		16 (Kurtosis)	0.227	0.721	0.651	0.707
	Euclidean	12	0.270	0.760	0.639	0.708
		16	0.270	0.760	0.642	0.709
	Manhattan	12	0.343	0.762	0.661	0.718
		16	0.328	0.782	0.675	0.720
SIMCOMP	(Maximal Common Subgraph)	-	0.400	0.847	0.779	0.808
EVA	-	σ = 100 cm^-1^	0.442	-	-	-
		σ = 50 cm^-1^	0.388	-	-	-
		σ = 20 cm^-1^	0.381	-	-	-
UNITY2D	-	-	0.247	-	-	-
MACCS	(Tanimoto)	166 bit key	0.364	0.778	0.659	0.742
MOLPRINT2D	(Tanimoto)		0.516	0.848	0.755	0.806
Atom Count	-		0.400	0.467	0.460	0.850

For the 3DZD, orders of expansion 4-14 have been applied and similarities calculated using the correlation coefficient, the Euclidean and the scaled Manhattan distances. For the USR the fourth statistical moment kurtosis has also been calculated. Hierarchical clustering was done using Ward's method. Results for the EVA and UNITY2D descriptors (for 7 clusters) have been taken from Table 2 in the article by Takane and Mitchell.

For the 3DZD, similarity matrices were built based on the three different measures of the correlation coefficient (r), D_E_, and D_M_. Orders of expansion ranging from 4 to 14 were tested, with significant gains observed in the value of the Rand index, as the order increases. The highest adjusted Rand index (0.487) is obtained for the 3DZD, where both correlation and D_E _metrics provide the same results for an order 12 expansion. The value becomes worse when the D_M _is used (0.393). Using a higher order (here we examined 14) resulted in smaller adjusted Rand index value which also suggests that expansion orders of 10-12 should be appropriate for comparison. The poorer performance at this level (>12) can be attributed to the noise resulting from far too detailed a description of the molecular shape. On the other hand, using a smaller order say 4-6, results in a much lower Rand index value (0.25-0.30), implying insufficient resolution for shape description.

The highest value achieved by the 3DZD (0.487) is higher than that of SIMCOMP (0.400), USR (0.342) and its variant USR-*k *(0.328), MACCS (0.364), and the atom count method (0.400). The 3DZD also marginally outperforms the EVA descriptor based classification which achieved a highest Rand index value of 0.480. However, Molprint-2D, which considers atom environments and atom types, shows the highest value among all (0.516).

The methods are also evaluated by two ranking-based scores, BEDROC and ROCAUC. The performance of the 3DZD becomes worse when evaluated by these two scores relative to the other methods. When evaluated by BEDROC (α = 160.9), Molprint-2D shows the highest value (0.848), and the rest of the methods are ranked in the following order: SIMCOMP, USR (Manhattan, 0.782), MACCS (0.778), and the 3DZD (0.739 with the correlation coefficient, order = 8). In terms of BEDROC (α = 32.2) and ROCAUC, SIMCOMP shows the highest value and Molprint-2D comes to the close second. With the AUCROC, the 3DZD (0.748 with Manhattan, order = 14) is ranked the third, and MACCS (0.742), USR (0.718 with Manhattan, order = 12) follow in this order. Although BEDROC and ROCAUC are frequently used ranking-based scores, it would be argued that such ranking-based scores are not very appropriate for a small dataset like the odour dataset [65]. As mentioned in Method section, BEDROC α = 160.9 and α = 32.2 emphasize ranks in top 1% and 5%, respectively, which correspond only to the top rank and top two ranks for the odour dataset of 47 compounds. On the other hand, the AUCROC computes unintuitively high value for many search results since the number of hits in the dataset is relatively high (5 to 11 hits among 49 total, as described in the dataset section).

**Figure 2:**
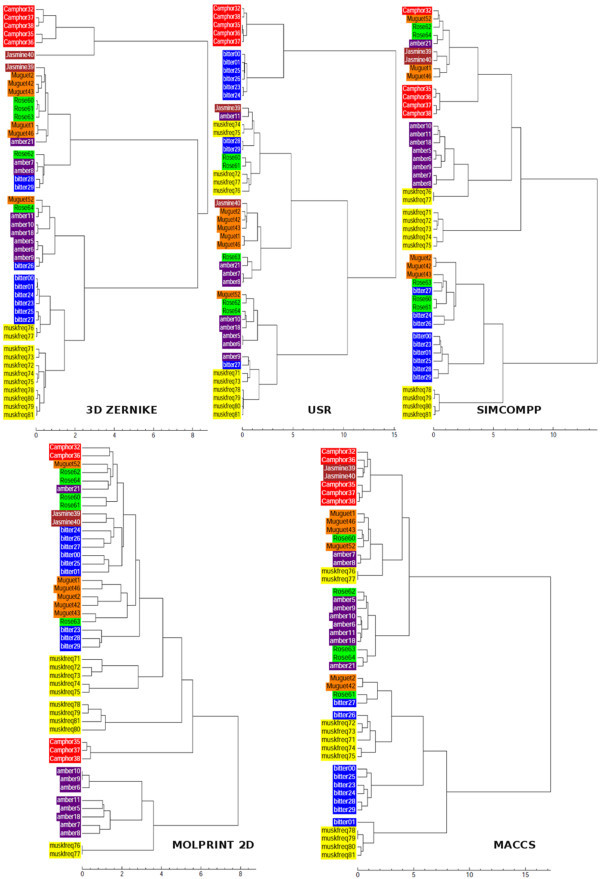
**Dendrograms using Ward's method are shown for 3DZD, SIMCOMP, USR, Molprint-2D and MACCS**. Each type of the compounds are differently colored.

Dendrograms for the five methods (3DZD correlation, USR Manhattan, SIMCOMP, Molprint-2D, and MACCS) are shown in Figure 2. None of the methods provide perfect distinction between the compounds and they produce different groupings. For the 3DZD based clustering, all camphor compounds are located in a separate partition while the musk odours are placed in two neighbouring clusters. Although USR manages to separate the camphor structures from the rest, the bitter almond series are located in three different groups (a trait shared by the 3DZD as well). On the whole, however, a poor separation of the other odours is seen, resulting in a smaller adjusted Rand index (0.343). SIMCOMP places most of the amber compounds in the same cluster, but splits the camphor series into two groups. On the other hand, the EVA descriptor based clustering (see Table 3 in the reference [43]) was able to locate the amber compounds in a single cluster; the other fragrances were split across multiple partitions. Molprint-2D classifies muguet compounds in a single cluster and also well captures similarity of amber compounds. Finally, MACCS shows a slightly different clustering, capturing similarity of camphor compounds but considers jasmine similar to camphor.

**Table 3:** Summary of the performance of the methods in the DUD dataset.

	EF 5%	BEDROC (α = 32.2)	AUCROC
3DZD (order 12, Correlation coeff.)	2.90	0.14	0.59
USR (order = 16, Correlation coeff.)	2.99	0.12	0.62
MACCS	4.22	0.23	0.52
Atom Count	1.37	0.07	0.34

The average value of the methods are shown.

**Figure 3:**
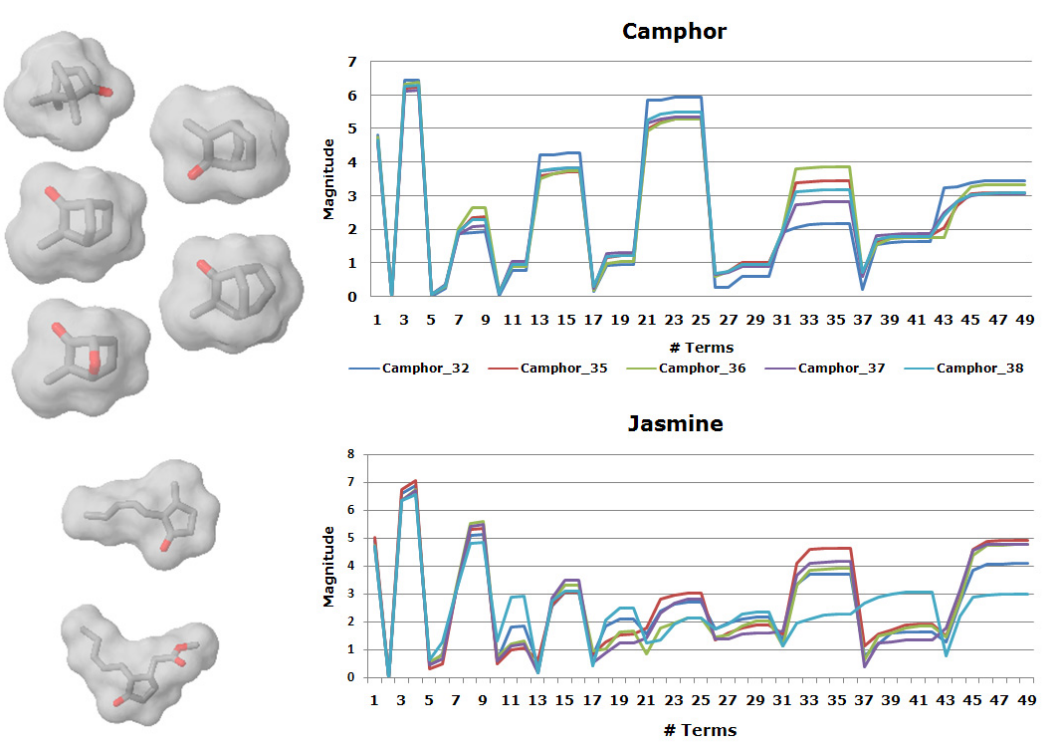
**The graph shows the comparison of the 3DZD invariants for the camphor and jasmine fragrances**. The curves depict the magnitudes of the moments and have plotted for an order 12 expansion. Similarity of the invariants for odour molecules of the camphor class explains why the 3DZD clusters all the camphors together. The two jasmine compounds however have different magnitudes for some of the moments, and result in a smaller similarity value (the correlation coefficient of 0.78).

While most of the methods clustered the camphor structures in the same group, the two jasmine fragrances are typically split. The 3DZD places one of the jasmines (Jasmine_40) by itself in a separate cluster. Inspection of the 3DZD for these compounds shows that their magnitudes follow very different trends. Graphs of the invariants are shown for camphor and jasmine (Figure 3), the former illustrating the similar trend in the values (3DZD for the camphor series) with all the compounds placed in a single group. In contrast, using Jasmine_39 as the query tends to pick up amber, muguet, and bitter compounds as the top 3 hits with corresponding correlation coefficients of 0.990, 0.988, and 0.987, respectively. The other jasmine compound (Jasmine_40) yields a much lower correlation value of 0.782 and is ranked poorly at 41.

Overall, the clustering results based on the 3DZD for the odour dataset have been encouraging. To further evaluate this approach, we have analysed the performances with respect to much larger datasets, results for which are presented in the next section.

### DUD dataset

In the previous section, we investigated how well the methods classify compounds using a small dataset of 47 odour compounds. Next, we use a larger dataset, namely, the DUD dataset (Table 1), to examine the performance of the methods in ranking and retrieving active compounds. The DUD dataset is appropriate for this task since it is developed for virtual screening benchmark and has been used in several previous studies. For the query, the active ligand molecule crystallized with the target protein is used to retrieve the other known active molecules among decoys. We compare 3DZD, USR, MACCS, and the atom count method. Three ranking-based evaluation metrics are used, *i.e. *BEDROC (a = 32.2), the enrichment factor (5%), and the AUCROC.

**Figure 4:**
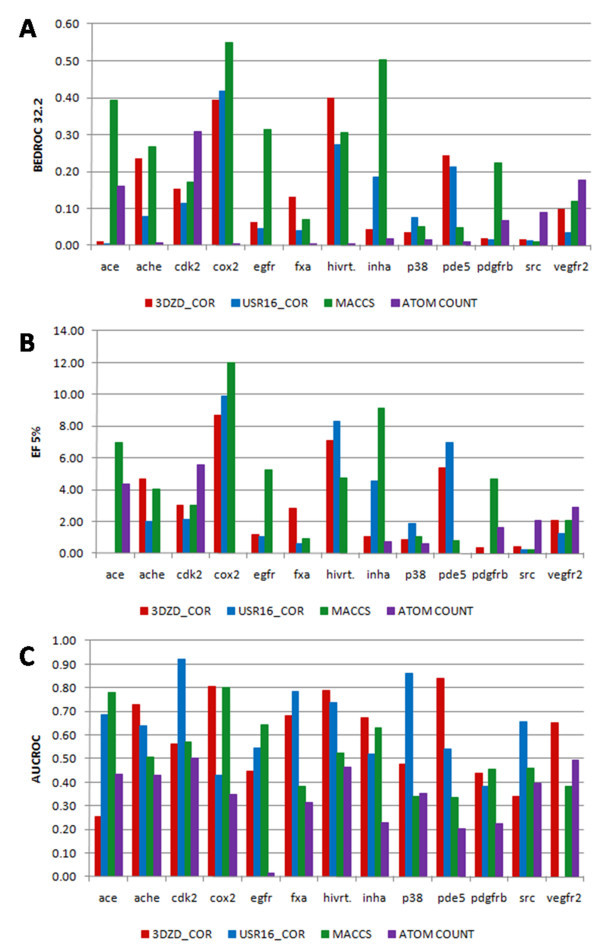
**Performance of the 3DZD, USR, MACCS, and the atom count methods on the 13 targets of the DUD dataset (Table 1)**. Three evaluation metrics are used: **A**, BEDROC (α = 32.2); **B**, EF5%; **C**, AUCROC. For the 3DZD, the order of 12 and the correlation coefficient is used as the distance metric. For the USR, the order of 16 and the correlation coefficient is used as the distance metric. The color code of the bars: red, 3DZD; blue, USR; green, MACCS; and purple, Atom count.

Figure 4 shows the performance of the four methods for each target. In addition, the summary (the average values) are provided in Table 3. On average the three methods, 3DZD, USR, and MACCS, all outperform the atom count method in all three evaluation metrics (Table 3), although there are some individual targets where the atom count method performs better than the others when evaluated by certain metrics (for example, results for vegfr2 and src evaluated by BEDROC and EF5%). The rankings of the three methods are not consistent across different evaluation metric used. When EF5% is used, MACCS shows the highest score and the 3DZD and USR follow in this order. Using BEDROC, MACSS stays at the best rank and the order of the 3DZD and USR switches. On the other hand, USR shows the highest score in terms of AUCROC and 3DZD comes to the second. These results illustrate difficulty of evaluating virtual screening methods and importance of evaluating methods by using several different metrics.

### NCI anti-HIV dataset

The third dataset, the anti-HIV dataset from the National Cancer Institute, is employed to simulate a typical virtual screening experiment. With both the actives and inactives forming the database to be searched, each of the remaining 904 moderately active molecules was used as the query. USR typically takes about 0.74 (12 terms)-0.76 (16 terms) seconds per query. Timings for the 3DZD comparisons are about 3 times that of the USR with per query comparisons taking 2.62 seconds for order 8 expansions and up to 2.70 seconds for order 14. It must be noted that the 3DZD considers more terms ranging from 25 (order 8) to 64 terms (order 14). The timings are reported with respect for 38352 database molecules that were compared and include the calculation of all the three distance metrics (correlation, D_E_, and D_M_). In comparison, the graph based SIMCOMP is significantly slower with timings exceeding an hour (~4245 seconds/query) in most cases.

The relative performances of the different methods were assessed using the BEDROC scores, enrichment factors, and AUCROC, which are shown in Table 4. As with the odour dataset case, for the range of expansion orders of the 3DZD tested, there is a trend where higher order terms lead to increase the enrichment. This trend is clear in AUCROC.

**Table 4:** The enrichment factors, BEDROC, and AUC ROC scores evaluated for the anti-HIV dataset.

Method							
Descriptors	Metric	Order	EF1%	EF5%	BEDROC (α = 160.9)	BEDROC (α = 32.2)	AUC ROC
3DZD	Correlation coefficient	4	1.887	1.298	0.0241	0.0485	0.421
		6	1.996	1.334	0.0261	0.0500	0.423
		8	2.006	1.297	0.0260	0.0490	0.430
		10	1.932	1.208	0.0252	0.0461	0.435
		12	2.006	1.297	0.0260	0.0490	0.430
		14	1.796	1.146	0.0238	0.0440	0.444
	Euclidean (D_E_)	4	1.546	1.307	0.0199	0.0471	0.411
		6	1.634	1.292	0.0213	0.0473	0.416
		8	1.725	1.301	0.0225	0.0477	0.427
		10	1.737	1.255	0.0227	0.0464	0.435
		12	1.728	1.301	0.0226	0.0477	0.427
		14	1.723	1.263	0.0227	0.0470	0.455
	Manhattan (D_M_)	4	1.561	1.281	0.0199	0.0466	0.412
		6	1.643	1.267	0.0212	0.0463	0.418
		8	1.735	1.250	0.0224	0.0462	0.431
		10	1.742	1.201	0.0226	0.0448	0.442
		12	1.740	1.251	0.0224	0.0462	0.431
		14	1.720	1.222	0.0226	0.0461	0.463
USR	Correlation coefficient	12	1.778	1.248	0.0229	0.0461	0.417
		16	1.706	1.357	0.0222	0.0480	0.422
	D_E_	12	1.955	1.301	0.0256	0.0486	0.392
		16	1.983	1.296	0.0261	0.0485	0.386
	D_M_	12	2.057	1.403	0.0268	0.0515	0.395
		16	2.045	1.335	0.0268	0.0497	0.386
SIMCOMP	(Maximal Common Subgraph)	**-**	2.735	1.277	0.0383	0.0528	0.477
Atom Count	-	**-**	1.972	1.581	0.0248	0.0562	0.422

The performance of the 3DZD for this dataset is however slightly poorer in comparison to the other methods tested. SIMCOMP achieves the highest value for the enrichment factor value at the 1% cutoff (2.735), BEDROC (α = 160.9) (0.0383), and for AUCROC (0.477). It also shows a better score than the 3DZD for BEDROC (α = 32.2). USR (Manhattan, with the order of 12) outperforms the 3DZD at all the metrics except for AUCROC. However, the relatively small value by all the methods, the 3DZD, USR, and SIMCOMP, suggests that all the methods compared here would not effective from an early recognition perspective for the anti-HIV dataset. It is particularly notable that the performance of the atom count method is close to the other methods on this dataset and even shows the highest value in the EF5% and BEDROC (α = 32.2). These results imply that molecular shape information is not effective as it is for the previous two datasets. We discuss the nature of this dataset in Discussion, which could be a reason of the results.

To understand, why the three methods (3DZD, USR, SIMCOMP) have relatively poorer results for this dataset we examined the structures retrieved for specific queries. Three different cases are considered:

I. A case where the 3DZD is able to retrieve more hits in the top 100 than SIMCOMP and USR,

II. A case where SIMCOMP outperforms USR and 3DZD, and

III. Where USR does better than SIMCOMP and 3DZD.

**Figure 5:**
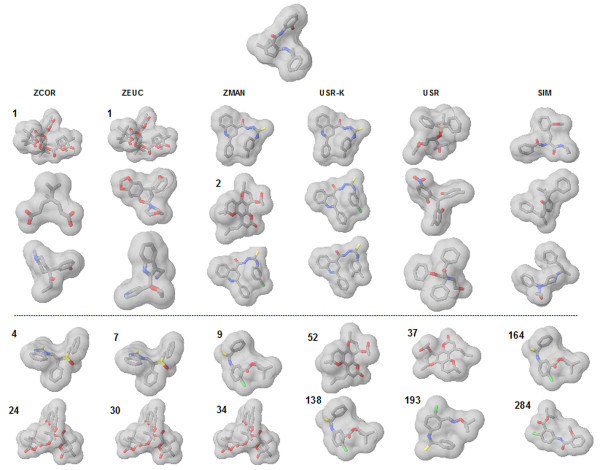
**Illustration of the case where the 3DZD retrieves more hits in the top 100 as compared to SIMCOMP and USR**. The query is indicated at the top of the figure. For each of the distance metrics used, five molecules are shown. The first three molecules in each column are the top ranked molecules. And the last two shown in the fourth and the fifth rows are two next highest ranked active molecules. Active molecules are identified as those with numbers (in bold) which indicate the ranks. ZCOR, ZEUC, and ZMAN, 3DZD using the correlation coefficient, D_E_, and D_M_, respectively; USR-K and USR, USR with/without kurtosis; SIM, SIMCOMP.

The first case is illustrated by the example in Figure 5 that lists five structures retrieved of which two are active. It can be seen that the 3DZD is able to retrieve actives and rank them much higher than USR and SIMCOMP. All three similarity metrics - the correlation coefficient, D_E_, and D_M _are able to discriminate almost equally well finding at least 2 actives in the top 10. Although difficult to generalize based on some of the cases considered, it would seem that the 3DZD, being an extension of spherical harmonics, is able to discriminate symmetrically shaped structures well and is therefore more effective in retrieving actives for molecules of this class.

**Figure 6:**
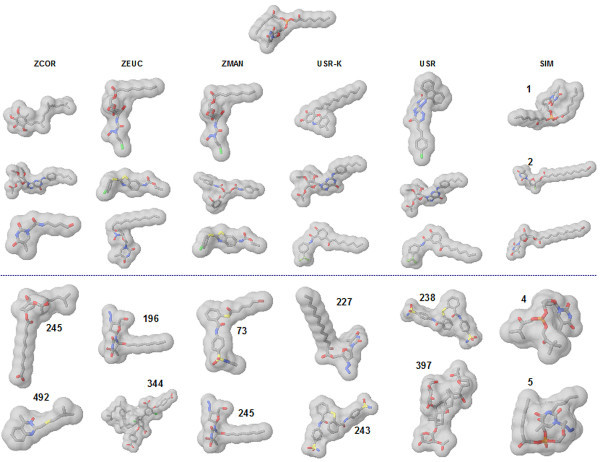
**Figure illustrates the case where the SIMCOMP performs better than 3DZD and USR in retrieving more actives in the top 100**. The query is indicated at the top of the figure. Active molecules are identified as those with numbers (in bold) which indicate the ranks. The first three molecules in each column are the top ranked molecules and the last two molecules are active molecules found in the two next highest ranks. Detailed information about the molecules is found at the lab web site.

Coming to the second case (shown in Figure 6), SIMCOMP achieves a much higher retrieval rate as compared to the other two methods. While on one hand these actives are retrieved very early on (4 out of top 5 are actives), the structures are quite diverse, which is a useful feature to have for scaffold hopping (structures with similar bioactivity but different chemotype).

**Figure 7:**
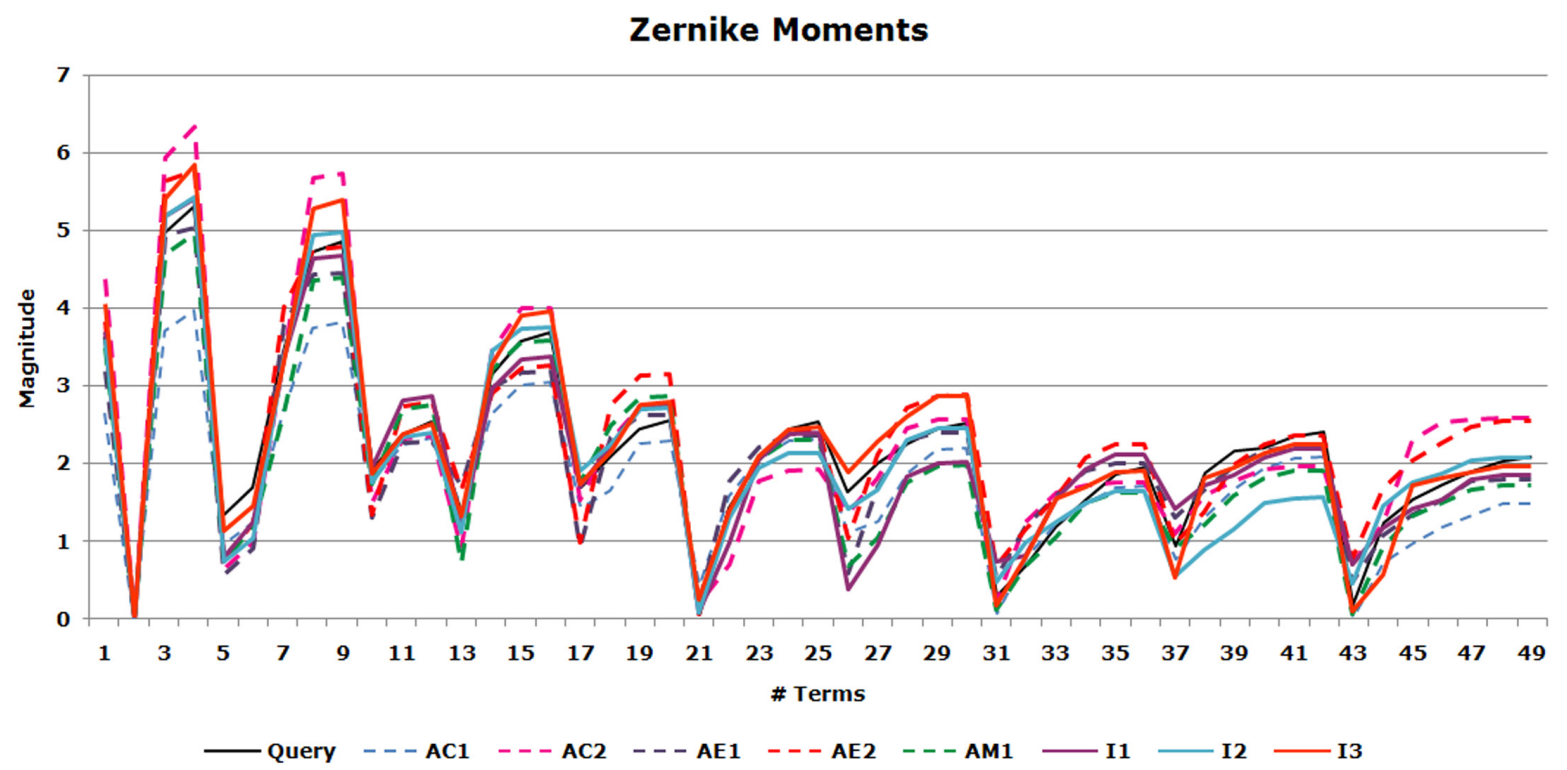
**Figure shows the plot of the 3DZD invariants for the case where SIMCOMP outperformed the two moment based methods**. The query is shown in black as solid line. I1, I2 and I3 refer to the top-ranking inactives ranked 1,2 and 3 and represented by solid lines. Those shown as dashed lines *i.e. *AC1, AC2 are the actives retrieved by the 3DZD correlation coefficient measure, AE1, AE2 the actives retrieved by the 3DZD (D_E_) and AM1 by the 3DZD (D_M_). Compared to the actives, moment invariants of the inactives seem to follow a more similar trend as compared to that of the query resulting in their being ranked at the top of the list.

In comparison, the other schemes find no actives in the top 100 structures with the exception of the 3DZD (D_M_) which finds a single active ranked 73. We therefore tried to analyze why the moment based methods behaved as they did. The graph shown in Figure 7 illustrates why this might be the case. It can be seen that the moments for the highest ranked actives (represented by the dashed lines) and inactives (represented by the bold lines) retrieved by the 3DZD for this case, follow the same trends as those of the query (shown in black). However, this trend is stronger for the inactives. Closer inspection of the correlation coefficients values (Table 4) of the 3DZD for the actives showed that their values were fairly close with difference of 0.0122 between the closest inactive retrieved (0.0995) and the first hit (active) ranked at 245 (0.9825). However, both D_E _and D_M _manifest this difference more clearly with values, thus the closest inactive is measured further closer relative to the first hit: the gap between the closest inactive and the first active is 0.7482 and 0.0575 for D_E _and D_M_, respectively. Thus, from a 3DZD point of view, these inactives are closer in the molecular shape to the query than the actives. SIMCOMP, on the other hand, using as it does the idea of atom environments, is able to capture similarities that the moment based methods missed (Figure 6).

**Figure 8:**
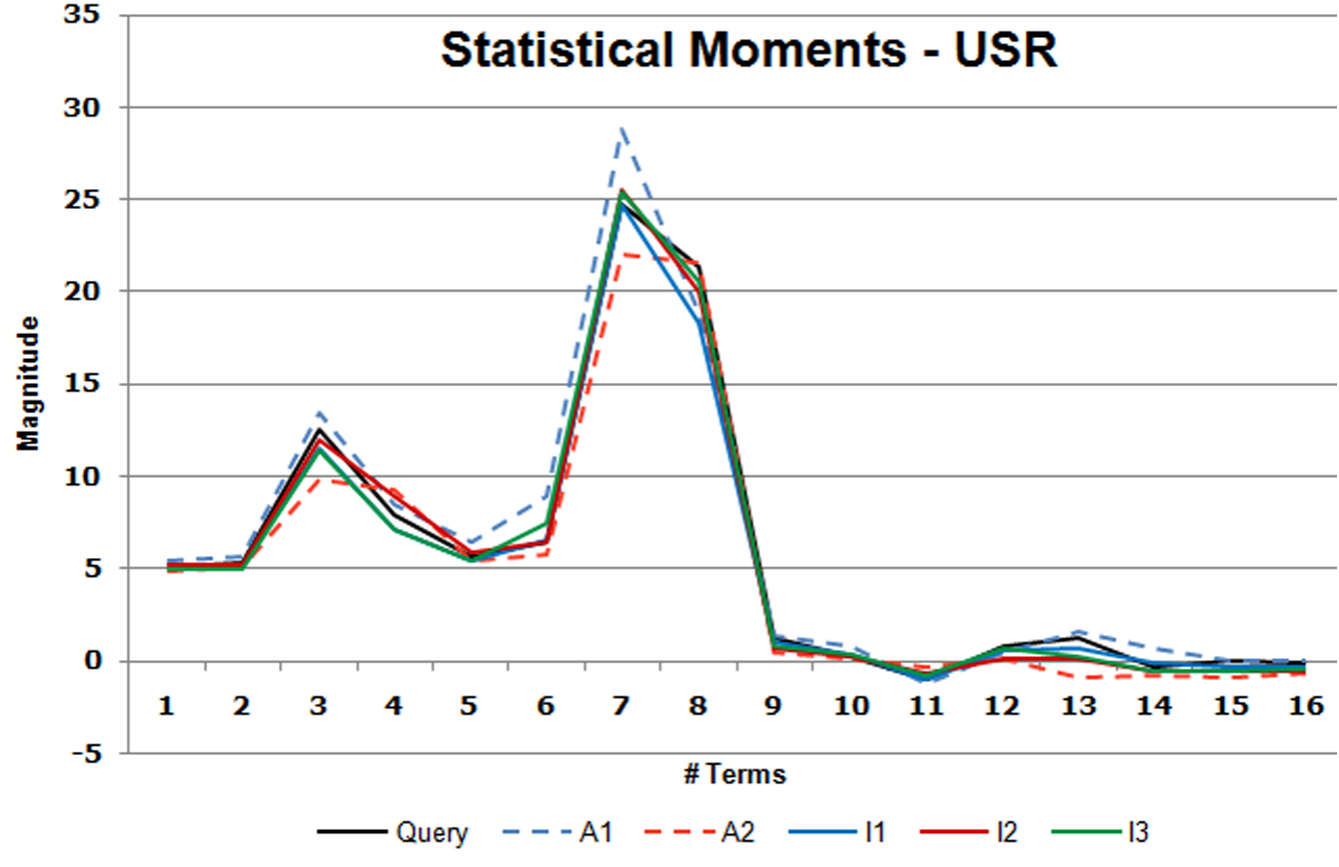
**Figure shows the plot of statistical moments generated by USR where the sixteen terms record the mean, variance, skewness and kurtosis of the interatomic distance distributions**. The query is shown in black as solid line. The three top ranking inactives (I1, I2, I3) are shown as solid lines while the actives A1 and A2 which were ranked much lower are shown as dashed lines. The plot serves as example for the case where SIMCOMP outperformed the two moment based methods. Moments of the actives show larger deviations compared to the inactives thus affecting the retrieval rates.

The statistical moments produced by USR were also examined in this context. Here also a pattern similar to that observed for the 3DZD is seen (Figure 8). While the moments of both the actives (dashed lines) and inactives (solid lines) trace the same trends as those followed by the query, greater deviations occur amongst the actives. Similarity scores with respect to the query are just above 0.5 (the left most column in Table 5) while those for the top 3 inactives average 0.66 with a relatively large difference of about 0.15 as shown in Table 5. This may suggest that, for cases where moments, both geometrical and statistical, are not as discriminating, other considerations are mandated - such as the atom environments used by SIMCOMP. This also reiterates the fact that while geometrical shape is a useful property to characterize molecules; it sometimes is by itself insufficient to provide a clear discrimination.

**Table 5:** Ranks and distance values for the case shown in Figure 5 where SIMCOMP outperforms both 3DZD and USR.

3DZD						USR-k		USR	
*Correlation*		*Euclidean*		*Manhattan*		*Manhattan*		*Manhattan*	
*Rank*	*Value*	*Rank*	*Value*	*Rank*	*Value*	*Rank*	*Value*	*Rank*	*Value*
1	0.9947	1	1.2152	1	0.8419	1	0.6752	1	0.6865
2	0.9918	2	1.2690	2	0.8376	2	0.6621	2	0.6732
3	0.9913	3	1.2969	3	0.8373	3	0.6620	3	0.6632
4	0.9825	196	1.9634	73	0.7844	227	0.5226	238	0.5281
5	0.9792	344	2.1038	245	0.7574	243	0.5208	397	0.5041

The top 3 structures retrieved are inactives with actives found much later shown in the fourth and the fifth rows.

**Figure 9:**
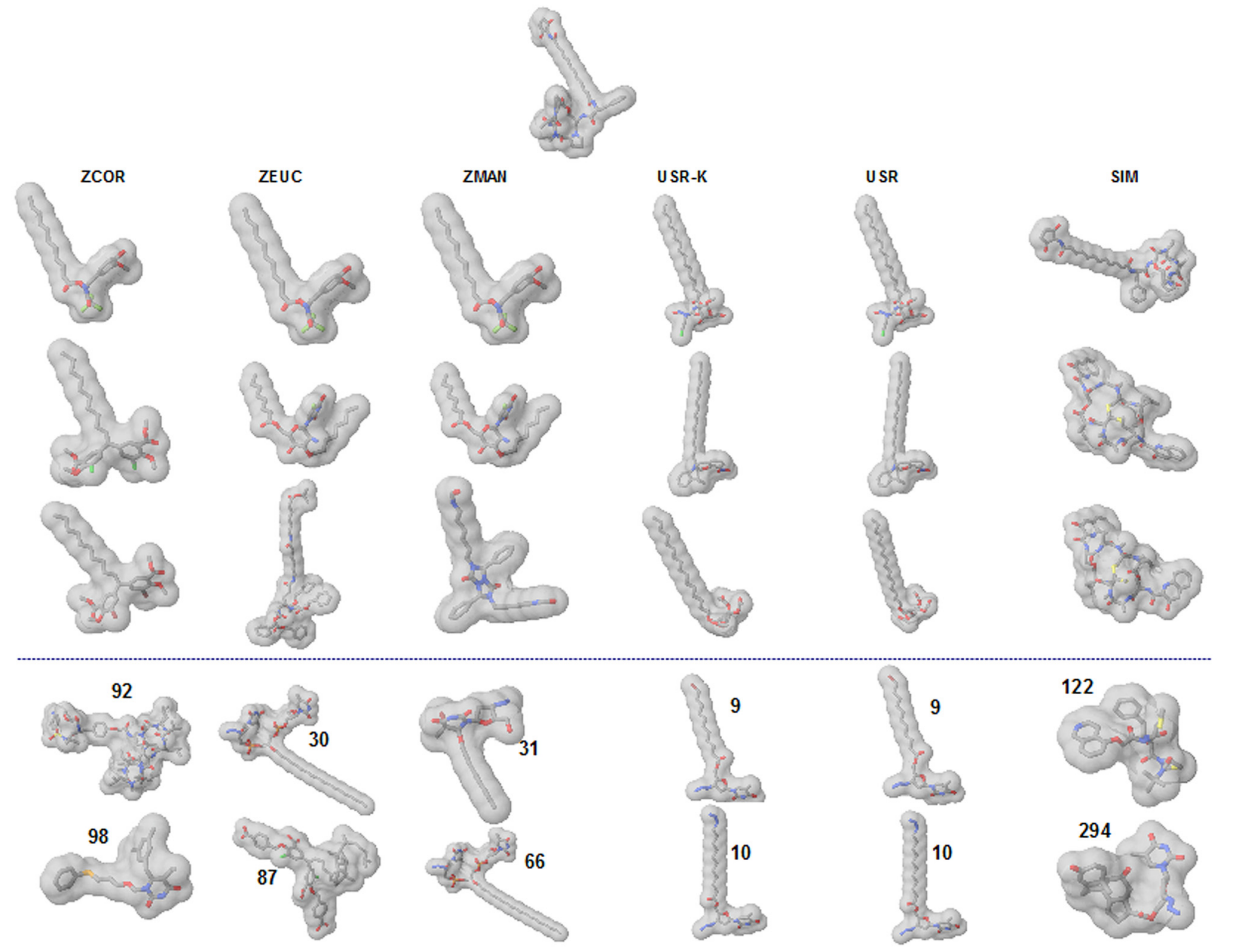
**Shown is the case where USR performs better than the 3DZD and SIMCOMP and retrieves more actives in the top 100**. The query is indicated at the top of the figure. Active molecules are identified as those with numbers (in bold) which indicate the ranks. The first three molecules in each column are the top ranked molecules and the last two are top ranked active molecules.

Finally, we analyzed the cases where USR reported a better discrimination of actives and inactives as compared to SIMCOMP and the 3DZD (Figure 9). For this query, the 3DZD does retrieve two actives within the top 100 while SIMCOMP obtains none. In contrast both USR and its kurtosis variant (USR-K) retrieve very similar looking structures [22] within the top 10. Though difficult to generalize based on these examples alone (Figures 5, 6 and 9) it is a case in point that USR may work relatively well for asymmetric molecules, considering as it has the third statistical moment, skewness, which is a measure of the molecule's asymmetry. On the other hand, the 3DZD seem to work better for spherical structures (Figure 6) rather than the other molecules that have typically elongated shapes and are not even close to being spherically symmetric (Figure 9).

On the basis of these results observed on the three datasets, it would be of interest to combine other molecular surface properties such as electrostatics and hydrophobicity that are captured implicitly by the atom environments used in SIMCOMP and Molprint-2D. While this may be a limiting factor for USR, the 3DZD facilitates their incorporation in a more convenient way as shown in our previous paper [40].

## Discussion

In this article, we have presented the 3D Zernike descriptors for ligand similarity searching. The work was motivated by previous studies that showed that these rotation invariant descriptors outperformed several other shape and alignment based comparison techniques when applied to proteins [38]. Compactness in terms of the number of coefficients generated in comparison to their spherical harmonics counterpart and easy extensibility to other molecular properties [40] were additional factors in favour of this descriptor. The compactness of the 3DZD enable fast comparison of compounds, which is a key property of virtual screening methods given the fast growing size of molecular databases.

The application to ligand similarity searching was exemplified using three datasets, each of which has a different purpose. The first odour dataset is suitable for examining ability to classify compounds into experimentally verified categories. The adjusted Rand index used as a measure of agreement with the known classification was found to be the second highest (0.487) for the 3DZD and it outperformed other shape based method (USR), a chemical graph matching scheme (SIMCOMP), a 2D finger print-based method (UNITY2D), the vibrational frequency based method (EVA), and MACCS. The second dataset, DUD, was chosen to investigate ability of ranking compounds, as the dataset has been used for the same purpose in several previous studies. All the methods compared consistently showed better performance than the simple atom count method, and the performance of the 3DZD was comparable among the methods.

For the third dataset, moderately active structures were used as queries to search an anti-HIV database of active and inactives. This dataset is intended to simulate actual application of virtual screening methods to a large pharmaceutical database. Rapid comparisons are facilitated by the floating point vector representation and both 3DZD and USR were found to be more than 60 orders faster than SIMCOMP. Although both USR and 3DZD describe shapes using moments, they exhibit a preference for specific shape types. Examination of a few cases suggests that the 3DZD may perform better for comparing molecules of more or less spherical shape while USR performs well for elongated asymmetric structures. However, a more detailed analysis of this would be required and is planned for the future. For this dataset, the 3DZD, USR, and SIMCOMP showed much lower values in terms of all the evaluation metrics as compared with the results for the previous two datasets and those typically seen in literature [62]. Moreover, the atom count method which simply considers the size of molecules showed comparable results, indicating molecular shape information did not add effective information for retrieval. To understand these results, it should be noted that this dataset may contain active compounds for multiple different molecular targets and, moreover, the heterogeneity of actives may be further increased due to the fact that the activity of compounds is measured by assays in living cell systems, where metabolism and uptake become important factor. Hence, generally speaking, we must say that this dataset is not among the most appropriate data for rigorous benchmark of virtual screening methods. In this study, however, we tried this dataset to mimic actual situation of the virtual screening after testing the methods on two well curated datasets.

Compound similarity searching by the 3DZD is intrinsically sensitive to the shape of molecules. This characteristic of the 3DZD can work as an advantage or can also lead to poor performance. In Figure 5, we showed a case that the 3DZD were able to find an active compound which was failed by SIMCOMP and USR. On the other hand, the two jasmine compounds in Figure 3 are the case where the 3DZD failed but the method which considers atom environments (*i.e. *SIMCOMP, see Figure 2), can detect their similarity. Figures 6 and 9 also exemplify compounds that the 3DZD find similarity based on the shape, which are not desired.

In summary, the 3DZD provide compact representations of molecular shape and can be applied to rapid screens of large compound databases. In addition to shape, other molecular properties can also be incorporated, thus enabling uniform comparison of the structures. The fact that they are surface based has other advantages such as comparison of shapes of binding pockets and the ligands bound to them, which are currently being studied in our group.

## Competing interests

The authors declare that they have no competing interests.

## Authors' contributions

VV implemented the USR method and scripts to perform database screening. VV and PR tested the presented methods and prepared the manuscript for this publication. DK conceived the study, supervised and coordinated the project, and helped writing the manuscript. All authors have read and approved of the final manuscript.
